# Nursing Care as Perceived by Nurses Working in Disability Community Settings in Greece

**DOI:** 10.5539/gjhs.v8n2p209

**Published:** 2015-06-25

**Authors:** Elpida Fotiadou, Maria Malliarou, Stella Zetta, Mary Gouva, Evaggelia Kotrotsiou

**Affiliations:** 1 Center of Welfare, District of Thessaly, “Aristeas Larissas”, Greece; 2 Technological Institution of TEI Thessaly, Greece; 3 Registered Nurse, Greece; 4 Technological Institution of TEI Ipeirou, Greece

**Keywords:** nursing care, nurses working, disability community, Greece

## Abstract

**Introduction-Aim:**

The concept of nursing care in learning disability community settings has not been investigated in Greece. The aim of this paper is to investigate how nurses working in learning disability community settings perceive the meaning of nursing care.

**Material and Methods:**

The sample consisted of 100 nurses and nursing assistants working in a social care hospice. Participants were asked to answer questions about socio- demographic characteristics of the sample and fill in a questionnaire of care (GR-NDI-24), the “Job-Communication-Satisfaction-Importance” (JCSI) questionnaire and the altruism scale of Ahmed and Jackson. The data analysis was realized with statistical methods of descriptive and inductive statistics. The analysis was made with the use of SPSS (version 19).

**Results:**

The majority of the sample was women (78%). The majority of participants were married (66 %), DE graduates (66%) without postgraduate studies (96.7%). The mean age of respondents was 36.98±6.70 years. On the scales of caring and altruism, the mean values were 40.89±15.87 and 28.12±4.16 respectively. Very or fully satisfied with his work was 72% of the sample. The scope of work emerges as the most important factor influencing job satisfaction. The wages and working conditions (73% and 40% respectively) are the parameters of work which gathers the most dissatisfaction, while the salary is emerging as the most important parameter, the improvement of which would provide the highest satisfaction. Marginally statistically significant difference was observed in the range between TE graduates (d=40) and those of the DE grade (d=37), p=0.053. No statistically significant differences were observed in relation to other working and demographic characteristics (p>0.05). Greater care importance was associated with greater job satisfaction (p<0.01), while the latter was associated with high levels of altruism (p<0.05).

**Conclusion:**

The scope of work provides high satisfaction to nurses working in social care hospices, while the salary is not satisfactory. Nurses’ aides appeared highly sensitive to care issues. A multidimensional approach to the materiality of care and job satisfaction in future research will allow to further highlight all the aspects affecting job satisfaction and performance of nurses. This will identify critical parameters of nursing care in healthcare centers for the chronically ill.

## 1. Background

Caring is fundamental to human experience and it is widely regarded to be central to the practice of nursing (Lea & Watson, 1996; Watson et al., 2003). Morrison and Bumard (1991) have documented the relationship between caring and nursing. Indeed caring has been widely affirmed as central nursing by Briggs (1972), McFarlane (1976), Watson (1979). According to Crowden (1994, p. 1106) “caring is a central and core element of nursing practice. Indeed, it may well be the essence of nursing”. In 1989 the Department of Health Nursing Division (p. 7) described nursing as “professional caring” whose fundament concern is the care and well-being of patients in a caring relationship the nurse as a person offers services to another person in a sense of the therapeutic relationship (A strategy for Nursing, 1989). The RCN definition of nursing refers to the ‘use of clinical judgement in the provision of care’ (Royal College of Nursing, 2003). Morse et al. (1991) provide five different conceptualizations of caring, all of which could adequately describe what nurses do. Therefore, while there is a distinction between, for example, the care a mother would give a child from that given by a nurse to a patient, the term caring - despite its own lack of definition is an adequate term to describe much of what nurses do. A single definition of caring is an unlikely prospect due to the multiple perspectives from which it is viewed and the range of actions which it encompasses (Morse et al., 1991). Caring is seen as a way of being and may or may not involve action or verbal communication (Noddings, 1984). It is through care, that the nurse observes and assesses the individual’s needs, implements the appropriate nursing interventions and makes informed decisions through encouraging positive change for the benefit of the patient (Clayton, 1991). Swanson (1991) defines caring as “a nurturing way of relating to a valued other toward whom one feels a personal sense of commitment and responsibility” (p. 162).

There has been some success in exploring the underlying dimensions of caring. Caring can be considered to have at least two dimensions and these have been described by James (1992) as the ‘physical labour’ and the ‘emotional labour’ of caring, and this is echoed by Clifford (1995) as the ‘instrumental’ and ‘expressive’ elements of caring. According to Noddings (1984) the process of caring is emotive rather than cognitive and concrete rather than abstract tied to a particular context. In addition, the nature of caring relationships can vary in intensity and may be episodic depending on the strength of felt obligation. During care, specific factors are involved. These include the relationship between the nurse - as the caregiver and the patient on the receiving end. Second factor is the personal care given which is a practical intervention and the nurse provides evidence of the ability to care. The third factor is the connection when the patient recognizes the value of the experience on offer. While the last factor of strengthening where the nurses and the patient enhanced, gain confidence from mutual experience to achieve specific results (Clayton, 1991).

Self-awareness in relation to feelings, attitudes, values and skills underlie the development of an effective caring relationship. The nurse recognizes the needs of the patient and his vulnerability. She responds to his needs with a fervent interest, patience and precision. Indulges in understanding the relief of the physical suffering, the emotional intensity and the need to maintain health (Hawks, 1992). The culture of environmental care is associated with trust, honesty, the open communication, kindness, interpersonal skills, mutual recognition and acceptance of people as they are. Along with all the required split vision, recognizing the value of change and politeness (Hawks, 1992).

From the perspective of health professionals, in order to provide care requires ethics, knowledge, art and experience. Also the prediction based on controlled events, identifying and meeting the needs of others, combinational thinking, sensitivity and skills implementation of plans to the welfare of others is essential (Streubert & Carpenter, 1999). Providing care to patients should be accompanied with the best clinical data, with the certainty that prevails against assumptions about the disease (Gortner & Schultz, 1993; Silva & Rothbert, 1984). The care that is based on scientific facts and evidence is illustrated through an epistemological diversity. It embraces many concepts and expressions based on four fundamental parts of knowledge: empirical, ethical, aesthetic and personal. The practice of providing care is complex and holistic, consequently knowledge based on more than one source, while as the source experience alone is inadequate in providing care (Blackburn, 1994). Perceptions of nurses about nursing care and patients’ expectations from nursing care are different McCance et al. (2009) has demonstrated that perceptions of nurses and patients regarding nursing care were congruent on statements related to technical and intimacy aspects of nursing.

Increasing demand for efficiency and quantity of production through an outdated industrial mindset has led to the distancing of patients from health professionals and among health professionals (Swanson, 1999). At the same time, the consciousness of nurses is changing and the main causes are the lack of personnel, the increased health system requirements and the lack of human care in personal-professional life in both systems and society. Nursing staff is among human values of care and the call of the profession increasingly oriented to the tasks of biomedical practice demands and heavy workload. The result according to Watson (2009) is a culture losing its way, a vacuum humanity for even greater increase in medical errors.

The CDI-25 has previously been used to demonstrate differences in the perception of caring between older and younger nurses and between men and women in nursing (Watson & Lea, 1998).

### 1.1 Aim

The relative absence of research data from Greece, which relate to the way in which nurses working in social care understand the meaning of nursing care have stimulated the need for this study. Therefore, the aim of the study is:

- To investigate how nurses working in a learning disability community setting perceive nursing care
- To investigate levels of job satisfaction among nurses working in a learning disability community setting
- To investigate levels of altruism among nurses working in a learning disability community setting

In addition we set the following research questions

1) Is there a correlation between how nurses perceive nursing care and the satisfaction that derive from their work?
2) Is there a correlation between how nurses perceive nursing care and altruism?

## 2. Methodology

The sample consisted of 100 nurses and nurses’ assistants working in different disability community settings of Thessaly and particularly the 15 nurses were from the Centre for Rehabilitation of Children with Disabilities of Karditsa (total of 15-over 100% participation), 25 nurses from the Chronic diseases Larissa “Aristeus” (100%) and 13 nurses from the Chronic disease Trikala (out of 19-percentage 68.5%). 57 questionnaires were distributed in two private institutions and 47 were returned completed. Nurses’ assistants help patients of all ages perform the most basic daily tasks. They work under a nurse’s supervision, and since they have extensive daily contact with each patient, they play a key role in the lives of their patients and in keeping the nurse up to date on vital information about the patients’ conditions.

### 2.1 Research Tools

The questionnaire consisted of four sections. The first section includes data on socio- demographic characteristics of the sample i.e. data on gender, age, marital status, educational level, place of residence, and employment status of the respondent. The second section involves a questionnaire on care which was adapted in Greek language by Kotrotsiou et al. (2014). The most commonly translation process for questionnaires or inventories which is the forward - backward translation (Yu, Lee, & Woo, 2004) was applied. The first step of this procedure involves a forward translation from the original language (English) to the language intended to be translated in (Greek). The second step includes back translation from the Greek to the original language (English) and consequently compared to the original version. A validation of the questionnaire was done by Kotrotsiou et al. (2014). The third section contains the questionnaire “Job-Communication - Satisfaction-Importance (JCSI)” The fourth and last section includes the altruism scale of Ahmed and Jackson (1979). More specifically, the three scales (caring, altruism and satisfaction) were as follows:

**Care Questionnaire (GR-NDI-24)**

The GR - NDI - 24 questionnaire of care is a self-assessment of perceptions of caring. There is a stem question (‘‘do you consider the following aspects of your nursing practice to be caring’’) and the respondent is required to indicate on a 5-point Likert scale ranging from 1 (very important) to 5 (not at all important) how they perceive caring (Kotrotsiou et al., 2014). The total score comes out of sum of all questions. Lower score corresponds to higher importance parameters of care. The internal reliability of the questionnaire in this study was high: Cronbach alpha=0.93.

**Job-Communication Satisfaction-Importance JCSI**

For the evaluation of the employment and communication satisfaction the questionnaire that was used was (Job-Communication Satisfaction-Importance (JCSI)), which was designed by Battey, 2010. The tool assesses communication in the field of nursing, the satisfaction that working can provide to caregivers and the importance of nursing. The responses of the tool can provide important information regarding whether healthcare personnel is properly trained in social skills in order to be able to communicate effectively with colleagues, superiors and patients. Additionally, it assesses the need for changes in the workplace and solve existing problems in order caregivers to work in a place that gives them satisfaction and care they provide to be highly effective and holistic [^30^]. The questionnaire JCSI consists of 28 items in which participants are asked to note the degree of satisfaction and importance, regarding their workplace. The evaluation is performed according to the representativeness of the content of the proposals for the subject, based on a seven-class type scale Likert [(-1) - (-2) - (-3) - (0) - (+1) - (+2) - (+3)]. The questionnaire has been adapted to the Greek language and presents sufficient Construct validity and satisfactory internal consistency reliability and test-retest reliability (Gouva et al., in press).

**Altruism Scale (Ahmed & Jackson, 1979)**

The dimension of altruism was measured using an adapted questionnaire, which was based on the questionnaire of Ahmed and Jackson. The questionnaire consists of eight items where the respondent answers on a likert scale 1 to 5 (Strongly disagree 1-5 absolutely agree) describing altruistic behavior. The total score is calculated by adding the scores of the responses to the seven questions, with higher values corresponding to higher levels of altruism. This questionnaire has been used in Greece and has adequate internal reliability (a=0.79) (Papageorgiou, 2009).

### 2.2 Procedure

The investigator informed employees in institutions and proceeded to describe the nature and purpose of the investigation, stating the ability to accept or to refuse to participate in research or even withdraw during the course of the study. In addition, another objective of this communication, which had an average duration of 20 minutes, was to create a safe and secure environment and a climate of trust. In the event that the employee agreed to take part in the study he/she could indicate a convenient meeting with the investigator to complete the questionnaire. During the meeting, the interviewer, giving questionnaires, provided a clear explanation for the entire process.

### 2.3 Ethical Considerations

This research study meets the fundamental ethical principles, which govern the conduct of psychological research. Specifically:

1). Complied with complete confidentiality in respect of information relating to their subjects and safeguarded the safety of the material.
2). Patented the anonymity of the test.
3). The results will be used solely for the purposes of this study and only by this research group.

## 3. Results

The majority of participants were women (78%), married (66%), and secondary school graduates (66%). Mean age of our sample was 36.98 years old. One third of our sample were Registered nurses with a technological level of nursing education and the rest two thirds were nurses’ assistants with two-year studies of nursing education (Table 1). The mean level of satisfaction and level of importance are presented in table 2. Nurses are satisfied the most by the value of relationships with colleagues and service and they think most important formal communication.

**Table 1:** Sociodemographic data

	Men N(%)	Female N(%)	Total N(%)	Differences
	22 (22.0)	78 (78.0)	100 (100.0)	p-value
**Age**				
x±S.D.	35.32±7.69	37.45±6.38	36.98±6.71	t=-1.321, P=.190
**Marital status**				
Single	9 (40.9)	15 (19.2)	24 (24.0)	Fisher’s Exact Test=4.292
Married	11 (50.0)	55 (70.5)	66 (66.0)	P=.109
Divorced	2 (9.1)	8 (10.3)	10 (10.0)	

**Level of nursing education**				
Two-year of study (nurses’ assistants)	10 (45.5)	56 (71.8)	66 (66.0)	L.R =5.089
Technological institution	12 (54.5)	22 (28.2)	34 (34.0)	P=.024
**Professional experience**				
0-5 years	9 (40.9)	14 (17.9)	23 (23.0)	Fisher’s Exact Test =5.075
5-10 years	5 (22.7)	28 (35.9)	33 (33.0)	P=.253
10-15 years	6 (27.3)	23 (29.5)	29 (29.0)	
15-20 years	2 (9.1)	9 (11.5)	11 (11.0)	
More than 20 years	0 (0.0)	4 (5.1)	4 (4.0)	

Note: x=Mean, SD=Standard deviation, t=T -Test, L.R.=Likelihood Ratio.

**Table 2:** Level of satisfaction and level of importance

Parameters of satisfaction from work	Level of satisfaction	Level of importance

	MT	MT
Information	0.77	1.85
Information with objectives	0.94	1.96
Information for achievements	1.19	1.89
Information for policy	0.68	1.89
**Formal Communication**	1.74	**2.65**
Meaningful success	1.35	2.02
Challenges working	1.30	1.92
Enjoyment of work	1.70	2.30
Using skills	1.45	2.46
Provided experience	1.04	2.19
Relationship with colleagues	1.44	2.23
Support from colleagues	1.75	2.37
Friendliness of colleagues	1.46	2.24
**Value of relationships with colleagues and service**	**2.53**	1.84
Informal communication	1.22	1.82
Relationship with superior	1.91	2.47
Communication with a superior	2.00	2.41
Communication by a superior	1.95	2.49
Recognition by a superior	1.80	2.50
Understanding by a superior	1.76	2.41
Instruction by a superior	1.71	2.47
Communication with the Director	1.06	2.27
Communication between departments	0.98	1.99
Communication with other specialties	1.61	2.30
Communication with volunteers	1.27	1.80

On the scales of caring and altruism average scores were 40.89±15.87 and 28.12±4.16 respectively. The value of relationships with colleagues was the section that employees felt more satisfied (mean 2.53), while as most important parameter they identified “formal communication” (mean 2.65) (Table 3).

**Table 3:** Correlation between job satisfaction, caring and altruism

		altruism	Satisfaction from work
Care	Correlation Coefficient	-.162	-.414^**^
	p	.107	<0.001
	N	100	100
Altruism	Correlation Coefficient		.209^*^
	p		.037
	N		100

Satisfaction score was negatively correlated with the care. This means that putting greater importance in the care is associated with greater job satisfaction. Also satisfaction scale was positively correlated with scores on the scale of altruism. This means that higher levels of altruism associated with greater job satisfaction.

Marginally statistically significant difference was observed in the range of NDI among registered nurses (d=40) and enrolled nurses (d=37), p=0.053 (Table 4). No statistically significant differences were identified in relation to other employment and demographic characteristics and levels of care and altruism.

**Table 4:** Correlation between care (GR-NDI-24) in relation to demographic characteristics

GR-NDI-24	MEDIAN (IQR)*	STATISTICAL TEST	p-value
**Sex**			
man	37 (27-45)	Mann-Whitney	0.316
woman	41 (34-55)		
**Marital Status**			
single	40 (37- 53)	Kruskal - Wallis	0.155
married	35 (27-47)		
widowed	38 (26-45)		
**Educational Level**			
2 year of studies	37 (27-45)	Μann-Whitney	0.053
technological institution graduates	40 (34a-57)		
**Experience**			
0-5 years	40 (30-49)	Kruskal - Wallis	0.935
5-10 years	38 (29-47)		
10-15 years	35 (27-52)		
≥ 15 years	37 (29-45)		

* interquartile range (25^th^ -75^th^).

## 4. Discussion

It is worth mentioning that enrolled nurses seem to put greater importance to the overall care compared with registered nurses. Nurses attach moderate importance to the parameters of care associated with the spirituality of patients. This may explained in the context of harsh working conditions in centers for the chronically ill (both in public and private sector) and the nature of the illnesses of the patients who often require constant care with their daily biological needs. Spirituality is important for nurses and can direct their interventions (Ray & McGee, 2006), but the limited resources of hospitals and poor staffing significantly limit this possibility.

The main chronic diseases which are associated with the admission in a chronic healthcare facility are dementia and stroke. Mental disorders account for 48% of total admissions, 43% physical disorders and 8% social / emotional problems (Rensbergen & Nawrot, 2010). The complex health needs and often unpredictable course of these diseases, make particularly challenging their care, often in a hostile environment, particularly in terms of hospitalization conditions and available resources (crowding of patients with heterogeneous diseases, short-staffing and high nursing turnover). Such adversity increase dissatisfaction among nurses may lead to a vicious cycle of withdrawal from work and health burden of patients. The financial rewards of health professionals working in chronic care facilities combined with working conditions are a source of dissatisfaction from work. This lack of job satisfaction has an impact on patient care (McHugh et al., 2011).

The main defenses against professional dissatisfaction and burnout are the qualities of a nurse combined with altruism. These may interfere with nurses’ participation in acts and attitudes relating to the common good and the legacy ideals of Nursing. In the present study altruism and satisfaction correlated significantly (p<0.05), a relationship that constitutes an antidote to deterioration of care in times prevailing in the country. Clinical training is necessary, but without the feelings of tender altruism and empathy cannot lead to comprehensive care.

Although worldwide nursing assistants do not enjoy high income, often are single-parent families and come from minority populations, demonstrate higher satisfaction even from registered nurses (Castle, Degenholtz et Rosen, 2006). This finding may be attributed to the close contact they develop with their patients which may be linked to the nature of their work. This relationship leads to a sense of self-realization and social contribution. Despite the difficult working conditions in centers for the chronically ill staff states disproportionately high satisfaction scores, a finding attributed to the high matching of patients, which in the present study showed that ranks high among the issues related to the significance of work.

The finding of high satisfaction of nurses in the sample is rather unlike other studies where nurses report high levels of dissatisfaction. As the biggest source of satisfaction was reported to be the scope of the work, confirming the view that wants the characteristics of a job to be an important factor that contributes to job satisfaction (Locke, 1976).

In the current study, wages and working conditions were the main causes of dissatisfaction among nurses. In addition poor staffing and a preoccupation with non- nursing tasks may be responsible for the discontent reported in the current study, a finding seen in other studies. Indeed, the nursing staff of all wards remains far from the standards of advanced countries (Doiron, Hall, & Jones, 2008; Stanton, 2005; Malliarou, 2010). In addition, limited time for in-service education combined with limited opportunities contribute to the professional development of staff dissatisfaction (Laschinger & Havens, 1997; Zangaro & Soeken, 2007).

Dissatisfaction with wages, which may be mitigated in the public safety of permanence for those who have permanent employment observed in other studies in Greek public hospitals (Zygoulis, 2008), as well as internationally (Coomber & Barriball, 2007; El-Jardali et al., 2009; Gardulf et al., 2005; Pillay, 2009), although the range of dissatisfaction varies depending on the wages in each country. Also, when asked about what changes in their work would give them greater satisfaction, participants’ respond was the wages, a finding that reflects the rapid deterioration of the economic situation in the country in recent years. This response is similar to responses expected from developing countries, where resentment of wages is very intense and where very low wages and infrastructure problems leading to departure from the nursing profession (El-Jardali et al., 2009; Gardulf et al., 2005; Pillay, 2009; El-Jardali et al., 2009; Gardulf et al., 2005; Pillay, 2009; Vujicic et al., 2004). However, in developed countries, and until recently in Greece too, the financial rewards are an important issue only in the absence of other sources of satisfaction (Sharp, 2008; Kourakos et al., 2012)

## 5. Conclusions

The findings of the current study indicate that nurses contribute in care in spite of adverse working conditions. Antidote to adversity is close interpersonal relationships with colleagues and patients. The improvement of working conditions and financial rewards is a standing demand of nurses which may lead to the improvement of their working life and hence will raise the level of overall care. A multidimensional approach to the materiality of care and job satisfaction in future research will allow to further highlight all the aspects affecting job satisfaction and performance of nurses. This will identify critical parameters of nursing care in healthcare centers for the chronically ill.
